# Racial Discrimination and Multimorbidity Among Older Adults in Colombia: A National Data Analysis

**DOI:** 10.5888/pcd20.220360

**Published:** 2023-05-04

**Authors:** Carlos A. Reyes-Ortiz, Torhonda Lee, Adalberto Campo-Arias, Jose Mauricio Ocampo-Chaparro, John S. Luque

**Affiliations:** 1 Institute of Public Health, College of Pharmacy and Pharmaceutical Sciences, Florida A&M University, Tallahassee, Florida; 2 Now with Department of Graduate Public Health, College of Veterinary Medicine, Tuskegee University, Tuskegee, Alabama; 3 Programa de Medicina, Facultad de Ciencias de la Salud, Universidad del Magdalena, Santa Marta, Colombia; 4 Programa de Geriatría, Departamento de Medicina Familiar, Universidad del Valle, Cali, Colombia; 5 Grupo Interinstitucional de Medicina Interna, Departamento de Medicina Interna, Universidad Libre, Cali, Colombia

## Abstract

**Introduction:**

Multimorbidity is a prevalent worldwide problem among older adults. Our objective was to assess the association between life-course racial discrimination and multimorbidity among older adults in Colombia.

**Methods:**

We used data from the SABE (Salud, Bienestar y Envejecimiento) Colombia Study in 2015 (N = 18,873), a national cross-sectional survey among adults aged 60 years or older. The outcome was multimorbidity, defined as having 2 or more chronic conditions. The main independent variables were 3 racial discrimination measures: 1) everyday racial discrimination (yes or no), 2) childhood racial discrimination score (scored from 0 [never] to 3 [many times]), and 3) situations of racial discrimination in the last 5 years (scored from 0 to 4 as a sum of the number of situations [group activities, public places, inside the family, health centers]). Other variables were sociodemographic characteristics, diseases, economic or health adversity during childhood, and functional status. We used weighted logistic regression analyses to adjust for differences between groups.

**Results:**

Multivariate logistic regression models showed that multimorbidity was significantly associated with experiencing everyday racial discrimination (OR, 2.21; 95% CI, 1.62–3.02), childhood racial discrimination (OR, 1.27; 95% CI, 1.10–1.47), and the number of situations of racial discrimination (OR= 1.56; 95% CI, 1.22–2.00). Multimorbidity was also independently associated with multimorbidity during childhood.

**Conclusion:**

Racial discrimination experiences were associated with higher odds of multimorbidity among older adults in Colombia. Strategies to decrease life course experiences of racial discrimination may improve the health of older adults.

**Summary**

**What is already known on this topic?**

Studies that used US national databases found an association between discrimination and multimorbidity; these studies focused on adults and everyday discrimination measures.

**What is added by this report?**

This study is the first to use national data on an older population in a Latin American nation to investigate the relationship between racial discrimination and multimorbidity. We found additional racial discrimination measures associated with multimorbidity, including childhood racial discrimination and recent racial discrimination situations.

**What are the implications for public health practice?**

Early identification of exposure to racial discrimination would help to inform strategies for preventing multimorbidity.

## Introduction

Multimorbidity, the coexistence of 2 or more chronic conditions, is a common problem among older adults worldwide (1). Multimorbidity is associated with greater vulnerability to diseases or safety issues, less resistance to acute health threats, and elevated risk of death, disability, poor functional status, poor quality of life, and adverse drug events (1,2). Identifying risk factors or underlying causes would help in developing strategies for preventing multimorbidity. Multimorbidity is highly prevalent among older adults in Colombia, but its relationship with experiences of racial or ethnic discrimination has not been explored (3).

In Latin America, racial discrimination based on skin color is a societal problem deeply rooted in the history of the region, which placed European conquerors and their descendants at the top of a racial and class-based hierarchy and enslaved Africans and subjugated Indigenous peoples at the bottom (4). Perceived discrimination has been associated with various adverse health outcomes among older adults, such as poor self-reported health, increased symptoms of depression, poor memory, chronic diseases, functional limitations, slow walking, recurrent falling, and shorter telomere length (5,6). One study in Puerto Rico identified a mediating relationship for social class between skin color and blood pressure, so complex sociocultural processes are at work between socially defined racial categories and health status (7). More studies have examined the associations between racial discrimination and single health conditions or diseases than have examined the relationship between racial discrimination and multimorbidity (8–11). One study using the National Survey of American Life with a sample of 5,191 African Americans found that people who experienced everyday discrimination and major discriminatory events were significantly more likely than those who did not experience any discrimination to report all types of multimorbidity (physical, psychiatric, mixed, any) (8). In another study, among 3,570 African Americans, everyday racial discrimination was associated with the total number of chronic health problems (9). In yet another study, which used data from the National Latino and Asian American Study and the National Survey of American Life, a significant positive association was found between perceived discrimination and chronic pain only among Hispanic respondents , not other racial and ethnic groups; no association was found between discrimination and chronic cardiovascular or respiratory conditions (10). In a study focused on 2,554 Hispanic adults in the US, everyday discrimination was associated with a greater count of chronic diseases (11).

Considering the multiple physical and mental health effects of racial discrimination on the older adult population in Colombia, we hypothesized that racial discrimination (everyday exposure, childhood events, or recent situations) would be independently associated with multimorbidity after adjusting for potential confounding factors. This relationship might be explained because people who have experienced racial discrimination may be frail and have risk factors commonly associated with multimorbidity, such as poor functional status and low physical performance (6). Moreover, racial and ethnic discrimination interact in a syndemic way with other adversities and social inequalities that increase the possibility of becoming ill or dying (12). The objective of this study was to assess the association between several measures of racial discrimination and multimorbidity among older adults in Colombia.

## Methods

This study was a secondary analysis of data from the SABE (Salud, Bienestar y Envejecimiento) Colombia Study, a cross-sectional survey conducted in urban and rural areas in Colombia among adults aged 60 years or older. Participants provided informed consent in the original study, and the ethics committees of the University of Caldas and the University of Valle approved the study protocol (13). The de-identified data are publicly available for secondary analysis.

### Design

SABE Colombia used a probabilistic, multistage, stratified sampling design. The survey was based on the national master sample for country population surveys in Colombia. Data were collected from April through September 2015 through interviews conducted in participants’ homes. Response rates were 62% in urban areas and 77% in rural areas. The final sample, including 244 municipalities in all departments (like states in the US), consisted of 23,694 men and women aged 60 years or older (13). The structure of SABE Colombia was like the structure of the SABE surveys led by the Pan American Health Organization in 7 Latin American cities (14). A section on violence, abuse, or discrimination experiences developed for the Colombian context was added to the survey. Detailed information about the SABE Colombia study and the sampling method is available elsewhere (13).

### Participants

Participants were eligible to participate in the survey if they were aged 60 years or older, could communicate with the research team, and provided written informed consent. At the beginning of each interview, the potential participant was administered the Folstein Mini-Mental State examination, a simple test of cognitive function (15); individuals who had a total score of less than 13 (of a total possible score of 30) were interviewed by proxy. We excluded from analysis participants with responses by proxy (n = 4,690; 17.5%) because they could not answer questions about discrimination and another 131 participants with missing values. These exclusions led to a final analytic sample of 18,873 participants aged 60 or older.

### Outcome

The outcome variable for this study was multimorbidity, which was assessed by asking the respondent the question “Have you ever been told by a doctor or a nurse that you have . . . ?” for each of the following medical conditions: hypertension, diabetes, coronary heart disease, arthritis, stroke, chronic pulmonary obstructive disease, osteoporosis, a mental (nervous, cognitive, or psychiatric) problem, or cancer. These medical conditions were counted from 0 to 9. Multimorbidity was defined as the presence of 2 or more chronic conditions (1,2).

### Primary independent variables

The interview was administered to the participant in a separate room if they lived with another person. The leading independent variable was self-reported experiences of discrimination, assessed by 3 questions, modified from discrimination scales described by Williams et al (16) and Krieger et al (17) and adapted from national population surveys on aging in Latin America (18). The first question addressed everyday racial discrimination: “Have you felt rejected or discriminated against because of your race or ethnicity?” This is a 1-item variable, yes or no.

The second question addressed childhood discrimination events due to skin color: “Thinking back to your childhood and when you went to school and college, did you ever feel rejected, discriminated against, treated badly or unfairly because of your skin color?” Possible responses to this 1-item variable were never (coded as 0), rarely (coded as 1), sometimes (coded as 2), and many times (coded as 3) for a total score from 0 to 3, with a higher score indicating more discrimination. For sensitivity analyses, we defined any childhood racial discrimination as having any (≥1) of the 3 options of having an experience of racial discrimination (rarely, sometimes, many times). Any childhood racial discrimination event was coded as 1, and no childhood racial discrimination event was coded as 0. In the SABE Colombia study, this variable was specifically constructed for racial and skin color discrimination and separated from the section on adverse childhood experiences. Childhood discrimination experiences are a part of lifetime discrimination as a person ages and should be considered an expanded measure of adverse childhood experiences (6).

The third question addressed recent situations of discrimination due to skin color: “In the last five years, at some point, you have felt discriminated against or treated unfairly because of your skin color in the following situations: 1) In meetings or group activities, 2) In public places (such as in the street, squares, shopping centers or markets, recreational centers, and transportation), 3) Within your family, and 4) In health centers, clinics, or hospitals.” This was a 4-item variable. Each item was coded as 0 (never or rarely) or 1 (sometimes or many times). The total score was created by summing the 4 items for a score of 0 to 4, with a higher score indicating more discrimination. This measure has an internal consistency of 0.71 (Cronbach α). For sensitivity analyses, we defined any recent situation of racial discrimination as having any (≥1) of the 4 options. Any recent situation of racial discrimination event was coded as 1, and no situation of racial discrimination event was coded as 0.

### Other characteristics

We included established risk factors for multimorbidity among community-dwelling older adults that were available in the database (1,2) and education, race, and socioeconomic stratum (SES), variables considered relevant in previous discrimination studies (4). Sociodemographic variables were age (years), sex (male or female), marital status (married or not married), education (low, defined as 0–5 years or high, defined as ≥6 years), race (self-reported as social construct as Black, Indigenous, Mestizo [people of mixed ancestry with a White European and an Indigenous background], White, mixed, or other), place of residence (urban or rural), private health insurance (yes or no) (private or “contributive” indicates people who pay for their health insurance; other categories were subsidized, other, or none), and SES (1 = low/low, 2 = low, 3 = medium/low, 4 = medium, 5 = medium/high, and 6 = high [4, 5 and 6 were merged because of small numbers]. Stratum 1 comprises people who live in very low-income housing with little access to infrastructure (eg, sewage) and pay only 50% of the real cost of public services (eg, water, electricity). Stratum 6 comprises people living in high-income housing, with access to well-developed infrastructure or utilities; they pay up to 20% more than the real cost of public services (19).

The survey used the Lawton Instrumental Activities of Daily Living (IADL) Scale (20) to evaluate the functional status of the participant in 6 activities (using the telephone, taking medications, managing finances, preparing meals, shopping, and using transportation). Scores range from 0 to 6, with lower scores signifying lower functional status and a score of 5 or less considered low. Obesity was defined as a body mass index of 30.0 or more (calculated as weight in kilograms divided by height in meters squared and based on weight and height measured during the interview). Physical activity was assessed by the question, “Do you walk, at least three times a week, between 9 and 20 blocks (1.6 km) without resting?” Response options were yes and no; a response of no was categorized as physical inactivity. Smoking status was assessed as current or former smoker versus nonsmoker. Other childhood-related factors were also included: self-perceived childhood economic situation (poor or fair vs good, with poor considered childhood economic adversity) and self-perceived childhood health status (poor or fair vs good, with poor considered childhood health adversity). We counted from 0 to 7 the number of the following childhood diseases reported by the participant: asthma, bronchitis, hepatitis, measles, renal disease, rheumatic fever, or tuberculosis. Childhood multimorbidity was defined as the presence of 2 or more childhood diseases.

### Statistical analysis

We used complex survey analyses to weight data, adjusting for the sampling survey design. We calculated descriptive statistics such as percentages and means (SEs). We used Wald χ^2^ tests (categorical variables) and analysis of variance (continuous variables) in bivariate analyses of multimorbidity and independent variables. Multivariate logistic regression models examined characteristics associated with multimorbidity, and odds ratios (ORs) with 95% CIs were calculated. We combined expert knowledge with a data-driven variable selection method to explore the robustness of our models. We used the best subset selection method, based on bivariate *P* values below .25, for further extensive testing of the model according to Akaike information criterion (21). We also evaluated collinearity and excluded SES and childhood health adversity from models. Relevant interaction terms were tested. The level of statistical significance was set at *P* < .05. We used SAS version 9.4 (SAS Institute, Inc) for all analyses.

## Results

Study participants had a mean (SE) age of 68.4 (0.10) years; 53.6% were women, 45.9% were Mestizo, and 43.3% were classified as having a multimorbidity. For racial discrimination measures, 2.2% reported experiencing everyday racial discrimination, 4.6% reported experiencing childhood racial discrimination, and 3.1% reported experiencing any situation of racial discrimination in the last 5 years (Table 1).

**Table 1:** Demographic Characteristics of Participants (N = 18,873) in the SABE (*Salud, Bienestar y Envejecimiento*) Colombia Study, 2015

Characteristic	Value^a^
**Age, y**	
60–64	36.5
65–69	26.8
70–74	17.7
≥75	19.0
**Sex**	
Male	46.4
Female	53.6
**Race**	
Black	6.0
Indigenous	5.1
Mestizo^b^	45.9
White	30.5
Mixed	3.3
Other	9.2
**Marital status**	
Not married	44.3
Married	55.7
**Education, y**	
0–5 (low)	70.0
≥6 (high)	30.0
**Socioeconomic stratum**	
1 (low/low)	25.9
2 (low)	39.9
3 (medium/low)	26.8
4,5, and 6 (medium, medium/high, and high)	7.4
**Has private health insurance**	
Yes	51.9
No	48.1
**Place of residence**	
Urban	80.2
Rural	19.8
**Physical inactivity**	
Yes	42.2
No	57.8
**Smoking**	
Former or current	52.9
Never	47.1
**Obese^c^**	
Yes	21.1
No	78.9
**Functional status^d^**	
Low	12.6
High	87.4
**Childhood exposures**	
Self-perceived economic adversity	
Yes	66.0
No	34.0
Self-perceived health adversity	
Yes	19.3
No	80.7
Childhood multimorbidity^e^	
Yes (≥2 childhood diseases)	10.7
No (≤1 childhood diseases)	89.3
**Racial discrimination measures**	
Everyday racial discrimination^f^	
Yes	2.2
No	97.8
Childhood racial discrimination^g^	
Never	95.5
Rarely	1.2
Sometimes	1.7
Many times	1.6
Any childhood racial discrimination (rarely, sometimes, or many times)	4.6
Childhood racial discrimination score, mean (SE)^h^	0.09 (0.01)
Total no. of situations of racial discrimination in last 5 years^i^	
None	96.9
In meetings or group activities	2.0
In public places	0.5
Within your family	0.4
In health centers, clinics, or hospitals	0.2
Any situation of racial discrimination (any of the 4 previous options)	3.1
No. of situations of racial discrimination in past 5 years, mean (SE)^j^	0.05 (0.01)
**Outcome**	
Multimorbidity^k^	
Yes (≥2 conditions)	43.3
No (≤1 conditions)	56.7

a Unless otherwise indicated, values are weighted percentages.

b Defined as people of mixed ancestry with a White European and an Indigenous background.

c Defined as a body mass index ≥30.0, calculated as weight in kilograms divided by height in meters squared and based on weight and height measured during the interview.

d The Lawton Instrumental Activities of Daily Living Scale (20) evaluated the functional status of participants in 6 activities (using the telephone, taking medications, managing finances, preparing meals, shopping, and using transportation). Scores range from 0 to 6, with lower scores signifying lower functional status and a score ≤5 considered low.

e Survey asked about the following 7 childhood diseases: asthma, bronchitis, hepatitis, measles, renal disease, rheumatic fever, or tuberculosis.

f Question was, “Have you felt rejected or discriminated against because of your race or ethnicity?”

g Question was, “Thinking back to your childhood and when you went to school and college, did you ever feel rejected, discriminated against, treated badly or unfairly because of your skin color?”

h Scored from 0 to 3, with a higher score indicating more discrimination, based on following coding: never = 0, rarely = 1, sometimes = 2, and many times = 3.

i Question was, “In the last five years, at some point, you have felt discriminated against or treated unfairly because of your skin color in the following situations . . .”

j Each situation was coded as 0 (never or rarely) or 1 (sometimes or many times). Total score was created by summing the 4 items for a score of 0 to 4, with a higher score indicating more discrimination.

k Question was, “Have you ever been told by a doctor or a nurse that you have . . . ?” for each of the following medical conditions: hypertension, diabetes, coronary heart disease, arthritis, stroke, chronic pulmonary obstructive disease, osteoporosis, a mental (nervous, cognitive, or psychiatric) problem, or cancer. Multimorbidity was defined as the presence of ≥2 chronic conditions.

In bivariate analyses, all racial discrimination measures were significantly associated with multimorbidity. The following factors were also associated with multimorbidity: older age, female sex, not being married, low level of education, higher SES, having private health insurance, urban residence, physical inactivity, no history of smoking, obesity, low IADL score, childhood health adversity, and childhood multimorbidity (Table 2).

**Table 2:** Results of Weighted Bivariate Analyses, by the Outcome Multimorbidity, Among Participants (N = 18,873) in the SABE (*Salud, Bienestar y Envejecimiento*) Colombia Study, 2015^a^

Characteristic	Multimorbidity, % (n = 7,821)	No multimorbidity, % (n = 11,052)	*P* value^b^
**Age, y**			
60–64	35.0	65.0	<.001
65–69	43.4	56.6	
70–74	47.4	52.6	
≥75	55.1	44.9	
**Sex**			
Male	31.4	68.6	<.001
Female	53.6	46.4	
**Race**			
Black	42.7	57.3	.17
Indigenous	37.9	62.1	
Mestizo^c^	42.9	57.1	
White	45.8	54.2	
Mixed	44.4	55.6	
Other	39.7	60.3	
**Marital status**			
Not married	48.5	51.5	.007
Married	39.1	60.9	
**Education, y**			
0–5 (low)	45.3	54.7	.04
≥6 (high)	41.1	58.9	
**Socioeconomic stratum**			
1 (low/low)	38.9	61.1	<.001
2 (low)	43.1	56.9	
3 (medium/low)	46.9	53.1	
4,5, and 6 (medium, medium/high, and high)	46.2	53.8	
**Has private health insurance**			
Yes	47.4	52.6	<.001
No	38.8	61.2	
**Place of residence**			
Urban	45.1	54.9	<.001
Rural	35.7	64.3	
**Physical inactivity**			
Yes	54.5	45.5	<.001
No	35.1	64.9	
**Smoking**			
Former or current	38.8	61.2	<.001
Never	48.3	51.7	
**Obese^d^**			
Yes	57.2	42.8	<.001
No	39.6	60.4	
**Functional status^e^**			
Low	52.1	47.9	.002
High	39.6	60.4	
**Childhood exposures**			
Self-perceived economic adversity			
Yes	44.2	55.8	.29
No	41.5	58.5	
Self-perceived health adversity			
Yes	49.8	50.2	.003
No	41.7	58.3	
Childhood multimorbidity (≥2 diseases)^f^			
Yes (≥2 childhood diseases)	56.5	43.5	<.001
No (≤1 childhood diseases)	41.7	58.3	
**Racial discrimination measures**			
Everyday racial discrimination^g^			
Yes	58.5	41.5	.005
No	42.9	57.1	
Any childhood racial discrimination^h^			
Yes	55.7	44.3	.02
No	42.7	57.3	
Childhood racial discrimination score, mean (SE)^i^	0.13 (0.01)	0.07 (0.01)	<.001
Total no. of situations of racial discrimination in last 5 years, mean (SE)^j^	0.07 (0.01)	0.03 (0.01)	.03
Any situation of racial discrimination in last 5 years			
Yes	60.2	39.8	.04
No	42.7	57.3	

a Unless otherwise indicated, values are weighted percentages. Question on multimorbidity was, “Have you ever been told by a doctor or a nurse that you have . . . ?” for each of the following medical conditions: hypertension, diabetes, coronary heart disease, arthritis, stroke, chronic pulmonary obstructive disease, osteoporosis, a mental (nervous, cognitive, or psychiatric) problem, or cancer. Multimorbidity was defined as the presence of ≥2 chronic conditions.

b Determined by Wald χ^2^ tests (categorical variables) and analysis of variance (continuous variables); *P* <.05 considered significant.

c Defined as people of mixed ancestry with a White European and an Indigenous background.

d Defined as a body mass index ≥30.0, calculated as weight in kilograms divided by height in meters squared and based on weight and height measured during the interview.

e The Lawton Instrumental Activities of Daily Living Scale (20) evaluated the functional status of participants in 6 activities (using the telephone, taking medications, managing finances, preparing meals, shopping, and using transportation). Scores range from 0 to 6, with lower scores signifying lower functional status and a score ≤5 considered low.

f Survey asked about the following 7 childhood diseases: asthma, bronchitis, hepatitis, measles, renal disease, rheumatic fever, or tuberculosis.

g Question was, “Have you felt rejected or discriminated against because of your race or ethnicity?”

h Question was, “Thinking back to your childhood and when you went to school and college, did you ever feel rejected, discriminated against, treated badly or unfairly because of your skin color?”

i Scored from 0 to 3, with a higher score indicating more discrimination, based on following coding: never = 0, rarely = 1, sometimes = 2, and many times = 3.

j Question was, “In the last five years, at some point, you have felt discriminated against or treated unfairly because of your skin color in the following situations [4 situations listed, plus an option for zero].” Each situation was coded as 0 (never or rarely) or 1 (sometimes or many times). Total score was created by summing the 4 items for a score of 0 to 4, with a higher score indicating more discrimination.

In multivariate analysis, multimorbidity was significantly associated with everyday racial discrimination (OR, 2.21; 95% CI, 1.62–3.02) [Model 1], childhood racial discrimination score (OR, 1.27; 95% CI, 1.10–1.47) [Model 2], and number of situations of racial discrimination in the last 5 years (OR, 1.56; 95% CI, 1.22–2.00) [Model 3] (Table 3). Older age, female sex, low level of education, having private health insurance, urban residence, physical inactivity, obesity, low IADL score, and childhood multimorbidity were also independently associated with multimorbidity (Table 3). Sensitivity analyses also showed that any childhood racial discrimination (OR, 1.60; 95% CI, 1.18–2.18; *P* = .002) and any situation of racial discrimination (OR, 2.23; 95% CI, 1.30–3.83; *P* = .004) were independently associated with multimorbidity.

**Table 3:** Results of Weighted Multivariate Logistic Regression Analyses of Associations With Multimorbidity^a^ Among Participants (N = 18,873) in the SABE (*Salud, Bienestar y Envejecimiento*) Colombia Study, 2015

Characteristic	Odds ratio (95% CI) [*P* value]		
	Model 1^b^	Model 2^c^	Model 3^d^
**Age, y**			
60–64	1 [Reference]	1 [Reference]	1 [Reference]
65–69	1.39 (1.18–1.64) [<.001]	1.40 (1.17–1.66) [<.001]	1.39 (1.17–1.65) [<.001]
70–74	1.61 (1.32–1.96) [<.001]	1.61 (1.31–1.97) [<.001]	1.61 (1.32–1.97) [<.001]
≥75	1.93 (1.66–2.24) [<.001]	1.95 (1.66–2.28) [<.001]	1.95 (1.68–2.26) [<.001]
**Sex**			
Male	1 [Reference]	1 [Reference]	1 [Reference]
Female	2.00 (1.80–2.22) [<.001]	1.98 (1.78–2.20) [<.001]	2.00 (1.80–2.23) [<.001]
**Race**			
Black	1.08 (0.75–1.55) [.66]	1.13 (0.80–1.59) [.49]	1.12 (0.80–1.60) [.52]
Indigenous	1 [Reference]	1 [Reference]	1 [Reference]
Mestizo^e^	1.14 (0.80–1.63) [.41]	1.14 (0.82–1.59) [.44]	1.15 (0.81–1.63) [.44]
White	1.19 (0.85–1.68) [.31]	1.18 (0.86–1.62) [.32]	1.17 (0.83–1.64) [.37]
Mixed	1.30 (0.70–2.44) [.41]	1.26 (0.70–2.28) [.44]	1.21 (0.65–2.24) [.55]
Other	0.93 (0.62–1.41) [.74]	0.91 (0.61–1.35) [.63]	0.89 (0.59–1.36) [.60]
**Marital status**			
Not married	1 [Reference]	1 [Reference]	1 [Reference]
Married	0.93 (0.80–1.08) [.33]	0.93 (0.81–1.07) [.30]	0.92 (0.79–1.07) [.30]
**Education**			
High	1 [Reference]	1 [Reference]	1 [Reference]
Low	1.19 (1.03–1.37) [.02]	1.18 (1.02–1.36) [.03]	1.17 (1.02–1.35) [.03]
**Has private health insurance**			
No	1 [Reference]	1 [Reference]	1 [Reference]
Yes	1.41 (1.28–1.55) [<.001]	1.39 (1.25–1.53) [<.001]	1.41 (1.28–1.55) [<.001]
**Residence**			
Rural	1 [Reference]	1 [Reference]	1 [Reference]
Urban	1.44 (1.19–1.74) [<.001]	1.44 (1.19–1.75) [<.001]	1.43 (1.17–1.75) [<.001]
**Physical inactivity**			
No	1 [Reference]	1 [Reference]	1 [Reference]
Yes	1.68 (1.49–1.90) [<.001]	1.68 (1.48–1.90) [<.001]]	1.67 (1.48–1.88) [<.001]
**Smoking status**			
Never	1 [Reference]	1 [Reference]	1 [Reference]
Former or current smoker	0.88 (0.74–1.03) [.11]	0.88 (0.75–1.03) [.12]	0.87 (0.74–1.03) [.11]
**Obese^f^**			
No	1 [Reference]	1 [Reference]	1 [Reference]
Yes	1.76 (1.60–1.94) [<.001]	1.75 (1.59–1.94) [<.001]	1.77 (1.60–1.95) [<.001]
**Functional status^g^**			
High	1 [Reference]	1 [Reference]	1 [Reference]
Low	1.36 (1.19–1.55) [<.001]	1.36 (1.20–1.53) [<.001]	1.37 (1.20–1.55) [<.001]
**Childhood exposures**			
Childhood multimorbidity^h^			
No	1 [Reference]	1 [Reference]	1 [Reference]
Yes	1.86 (1.30–2.65) [<.001]	1.84 (1.28–2.63) [<.001]	1.85 (1.30–2.63) [<.001]
**Racial discrimination measures**			
Everyday racial discrimination^i^			
No	1 [Reference]	—	—
Yes	2.21 (1.62–3.02) [<.001]	—	—
Childhood racial discrimination score^j^	—	1.27 (1.10–1.47) [.001]	—
Total no. of situations of racial discrimination^k^	—	—	1.56 (1.22–2.00) [<.001]

Abbreviation: —, does not apply.

a Question on multimorbidity was, “Have you ever been told by a doctor or a nurse that you have . . . ?” for each of the following medical conditions: hypertension, diabetes, coronary heart disease, arthritis, stroke, chronic pulmonary obstructive disease, osteoporosis, a mental (nervous, cognitive, or psychiatric) problem, or cancer. Multimorbidity was defined as the presence of ≥2 chronic conditions.

b Racial discrimination is main predictor; covariates were adjusted for all variables in the table.

c Childhood racial discrimination is main predictor; covariates were adjusted for all variables in the table.

d Total number of situations of racial discrimination is main predictor; covariates were adjusted for all variables in the table.

e Defined as people of mixed ancestry with a White European and an Indigenous background.

f Defined as a body mass index ≥30.0, calculated as weight in kilograms divided by height in meters squared and based on weight and height measured during the interview.

g The Lawton Instrumental Activities of Daily Living Scale (20) evaluated the functional status of participants in 6 activities (using the telephone, taking medications, managing finances, preparing meals, shopping, and using transportation). Scores range from 0 to 6, with lower scores signifying lower functional status and a score ≤5 considered low.

h Survey asked about the following 7 childhood diseases: asthma, bronchitis, hepatitis, measles, renal disease, rheumatic fever, or tuberculosis.

i Question was, “Have you felt rejected or discriminated against because of your race or ethnicity?”

j Question was, “Thinking back to your childhood and when you went to school and college, did you ever feel rejected, discriminated against, treated badly or unfairly because of your skin color?” Scored from 0 to 3, with a higher score indicating more discrimination, based on following coding: never = 0, rarely = 1, sometimes = 2, and many times = 3.

k Question was, “In the last five years, at some point, you have felt discriminated against or treated unfairly because of your skin color in the following situations [4 situations listed, plus an option for zero].” Each situation was coded as 0 (never or rarely) or 1 (sometimes or many times). Total score was created by summing the 4 items for a score of 0 to 4, with a higher score indicating more discrimination.

## Discussion

We found that higher scores on multiple racial discrimination measures were significantly associated with higher odds of multimorbidity among adults aged 60 years or older in Colombia. This is one of the first studies on the topic that used a nationally representative sample of a country’s older adult population. Everyday racial discrimination, a higher childhood racial discrimination score, and a higher number of racial discrimination situations were significantly associated with multimorbidity after controlling for confounding factors.

Our results agree in part with other studies in the US that reported associations between racial discrimination and single health conditions or multimorbidity (8–11).

We consider that racial discrimination, within the larger construct of racism, represents cumulative stress and chronic psychological trauma during a lifetime (22), resulting in an additional risk factor for multimorbidity. Thus, discrimination as a source of chronic psychosocial stress results in neuroendocrine, autonomic, and immune systems dysregulation (23), which eventually results in changes in health outcomes conducive to multimorbidity.

In addition, the stress from racial discrimination has psychological consequences such as depressive symptoms and anxiety (22) that could lead to negative lifestyle and health behaviors, such as substance abuse, unhealthy diet, sleep problems, or physical inactivity (24,25), which together may lead to multimorbidity (2). Discrimination has also been associated with allostatic load (26), which as multisystem physiologic dysregulation and inflammation, predisposes a person to developing diseases such as hypertension and chronic kidney disease (27). Further research is needed to untangle these relationships to identify the independent effects of discrimination on multimorbidity.

An additional finding was the independent association between exposure to childhood multimorbidity and multimorbidity in older adults. This agrees with previous research findings where childhood disease has a direct negative association with later-life health (28). It seems that early-life conditions underlie susceptibility to later developing other diseases (28). Lower SES and poorer health conditions in childhood were associated with a greater likelihood of reporting physician-diagnosed heart diseases, even after controlling for conditions in adulthood and older age (29). A potential explanatory mechanism is the cohort morbidity phenotype hypothesis, where higher levels of infections at younger ages will be positively associated with inflammation and diseases at older ages because early infectious exposures may increase the activation of inflammatory pathways throughout the life course (30).

The research results mentioned (26–30) are closely related to a recent study that reported that a persistent exposure to racial discrimination predicted elevated inflammation and, in turn, chronic illnesses after adjusting for SES and other variables (31). Thus, the association between early and later multimorbidity in our study and other studies (28–30), along with our findings that early and later exposures to racial discrimination were associated with multimorbidity, may have a common pathway by chronic inflammation and allostatic load (26,30,31). Therefore, early interventions related to such exposures may reduce their health burden into older ages.

Our findings have potential implications for public health and medicine. All types of discrimination, such as everyday racial discrimination, childhood racial discrimination, and racial discrimination situations, reflect cumulative psychological trauma that may have late health consequences in older adults (32), such as multimorbidity. Indeed, the issue is complex, where racial discrimination, a frequent psychosocial risk factor, is associated with the biomedical multimorbidity syndrome and, from an aging perspective, could merit further attention from those who provide health care to older adults.

Concerning clinical practice, younger patients prone to experiencing discrimination should be referred to counselors or therapists who can help them mitigate the stress they may experience after being exposed to racial discrimination. This therapy may reduce long-term negative health consequences such as depression, poor self-rated health, recurrent falling, and multimorbidity (5,6,8,28,29,32).

This study has some limitations. The cross-sectional design did not allow us to determine causality or the direction of the relationship. Retrospective recall in the data collection may have caused recall bias. In addition, the discrimination questions are asked at older ages and not at early ages. Thus, people might self-select on their reporting (eg, those affected are more likely to report it), resulting in an upward bias, because we cannot observe those who experienced discrimination but were not similarly adversely affected. However, our study has several strengths. The study sample is representative of the older population in Colombia. We showed that several measures of racial discrimination are associated with multimorbidity, a pervasive geriatric problem. This supports the idea that repetitive discrimination throughout a person’s life may have later consequences in the development of multimorbidity.

Our findings open new areas of clinical and public health research by expanding the potentially harmful effect of lifetime racial discrimination exposure that should be considered in the pathway for multimorbidity.
